# Non-Invasive Three-Dimensional Cell Manipulation Technology Based on Acoustic Microfluidic Chips

**DOI:** 10.3390/mi16091068

**Published:** 2025-09-22

**Authors:** Lin Lin, Yiming Zhen, Wang Li, Guoqiang Dong, Rongxing Zhu, Minhui Liang

**Affiliations:** 1 School of Mechanical Engineering, Guangxi University, Nanning 530004, China; llin@gxu.edu.cn (L.L.); ex35680002ca@163.com (Y.Z.); 18692045737@163.com (W.L.); guoqiang20000221@163.com (G.D.); q1329728621@163.com (R.Z.); 2 Guangxi Key Lab of Manufacturing System and Advanced Manufacturing Technology, Nanning 530003, China

**Keywords:** acoustic microfluidic chips, three-dimensional manipulation, vibration modes, biological activity

## Abstract

This study presents a non-invasive three-dimensional cell manipulation technique based on acoustic microfluidic chips, which generates acoustic flow fields through the vibration of micropillars induced by bulk acoustic waves to achieve precise multi-dimensional rotational manipulation of cells. Moreover, the characteristics of the acoustic flow field under linear, quasi-circular, elliptical, and higher-order vibration modes were intensively studied, and the rotational manipulation performance of polystyrene microbeads and cancer cells was optimized by adjusting the frequency and voltage. The results showed that the rotational speed and direction of the particles varied significantly in different vibration modes, with the particles and cells achieving the highest rotational speed in the elliptical vibration mode (frequency: 44.9 kHz, and voltage: 60 Vpp). In addition, the technique successfully achieved in-plane and out-of-plane rotation of cancer cells, and cell viability tests showed that 94% of the cells remained active after manipulation, demonstrating the low damage and biocompatibility of the method. This study provides a new, efficient, precise and gentle approach to three-dimensional manipulation of cells, which holds significant potential in biomedical research and clinical applications.

## 1. Introduction

Cell rotation has broad implications in modern biomedical research and applications, covering a wide range of fields from basic research to clinical applications. By precisely controlling the rotation of cells, researchers can better understand the structure and function of cells, develop new diagnostic and therapeutic methods, and advance tissue engineering and regenerative medicine. Cell rotation can achieve multi-angle cell imaging [1,2,3,4] to eliminate blind spots in three-dimensional (3D) imaging and provide more comprehensive information about cell structure; cell rotation is crucial in cell surgery and micromanipulation [5,6,7,8,9,10], as precise control of cell rotation can reduce damage to the internal structure of cells. Cell rotation can also be used to characterize [11,12,13,14,15] cell properties for studying mechanical, electrical, and physiological states of cells, and can be used for drug research and tumor heterogeneity analysis [16,17,18,19]. By rotating cells, researchers can observe the effects of drugs on cells and analyze the heterogeneity of tumor cell populations.

The methods of cell rotation can be divided into contact manipulation and field manipulation. Contact manipulation includes mechanical contact and optical tweezers manipulation (OTs) [20]. The mechanical contact method involves directly touching the cell with a micromanipulator (such as a micropipette or microgripper) and rotating the cell through friction or thrust. For example, Wang et al. used a micropipette to rotate embryonic cells by friction, with an average rotation Angle error of 0.3°, a maximum Angle error of 0.5°, and a success rate of 92.5% [21], but this manipulation method may cause physical damage [22] to the cells, is operationally complex, requires highly skilled operators, and is not suitable for tiny cells (<100 μm) [4]. OTs use optical tweezers to capture cells through a highly focused laser beam and use the momentum transfer of the laser beam to make the cells rotate [9,10]. This manipulation method can avoid contact-induced cell damage, has a relatively high success rate of the system with a standard deviation of less than 3% [9,10,23,24] of the rotation rate. However, if high-power laser beams are used in the method of manipulating light fields [25], it may cause photodamage to cells [26] (such as local overheating [27] and protein denaturation [28]). If the output force of the laser beam is weak, it is difficult to rotate larger cells (such as oocytes) [29], and the equipment is expensive, the operation is complex, and it does not have selectivity for the manipulated particles [30]. Although contact manipulation methods perform well in specific scenarios, the potential risk of cell damage and the complexity of manipulation have prompted researchers to explore non-invasive field manipulation methods to provide more flexible and safer cell rotation options.

Field manipulations include electric field manipulations, magnetic field manipulations, acoustic field manipulations, and hydrodynamic field manipulations [20]. The electric field method involves rotating cells in a non-uniform electric field through dielectrophoresis (DEP) and electrorotation (ROT). This rotation method can be applied to cells of various sizes (0.001–1000 μm), and it has a low cost and high efficiency [31,32,33]. However, the method of manipulating through an electric field can produce side effects such as Joule heat [34], affect cell viability, and typically only achieve single-degree-of-freedom rotation, making it difficult to achieve multi-degree-of-freedom control [35]. Magnetic manipulation involves attaching magnetic microbeads to the cell and applying torque to the microbeads through an external magnetic field to rotate [36,37,38] the cell. This method is a non-invasive operation that avoids physical damage to the cells, but the magnetic field manipulation method requires pre-labeling of the cells, which may affect the physiological properties [39,40] of the cells, is difficult to precisely control the rotational speed [41], and usually only achieves a single degree of freedom of rotation [42,43]. Sound field manipulation involves generating acoustic radiation forces through sound waves, such as surface acoustic waves (SAWs) or volume acoustic waves (BAWs), to rotate cells. This method can be applied to cells of various sizes and can manipulate multiple cells with less [44,45,46,47] damage to the cells. The existing methods for manipulating cells with sound waves are difficult to achieve precise control and three-dimensional multi-degree-of-freedom rotation of cells [48]. Although the use of surface acoustic waves for cell manipulation can ensure the accuracy and freedom of manipulation, the preparation cost of piezoelectric substrates (such as lithium niobate) and interdigital transducers (IDTs) in the equipment is relatively high. The hydrodynamic field manipulation method generates a rotational force through the flow of fluid in a microfluidic device to drive the cell to rotate. For example, cell rotation is achieved through micro-vortices or vibration-induced fluid flows. This manipulation method is low-cost, easy to operate, suitable for multiple types of cells, and causes less [49,50,51,52,53] damage to the cells, but the method of manipulating cells using hydrodynamic fields is difficult to precisely control the rotational speed and direction [54,55]. It is suitable for high-throughput experiments, but has lower manipulation accuracy for individual cells.

Therefore, in order to overcome the shortcomings of the aforementioned manipulation methods, this study proposes an acoustic microcolumn array (AMPA) chip based on BAW control. The device uses piezoelectric transducers (PZT) to generate BAW, which have a simple structure and are low-cost. Furthermore, in our experiment, the control frequency of BAW (kHz) is much lower than that of SAW (MHz), causing less damage to cells. It utilizes the vibration of micropillars induced by BAW to generate an acoustic flow field, and precisely rotates and manipulates cells in multiple dimensions with the force of the acoustic flow field. The vibration mode of the sound waves has a significant impact on the flow characteristics of the acoustic flow field. We focus on the characteristics of the acoustic flow field in linear, quasi-circular, and elliptical vibration modes. In different vibration modes, the motion state of polystyrene particles in the acoustic flow field was observed by adjusting the frequency and voltage, the rotational maneuverability of the micro-particles in the acoustic flow field was evaluated, and the above research results were used for the study of cell manipulation. Finally, we investigated the impact of this manipulation method on cell viability.

## 2. Materials and Methods

### 2.1. AMPA Chip Composition and Manipulation Working Principle

**Figure 1:**
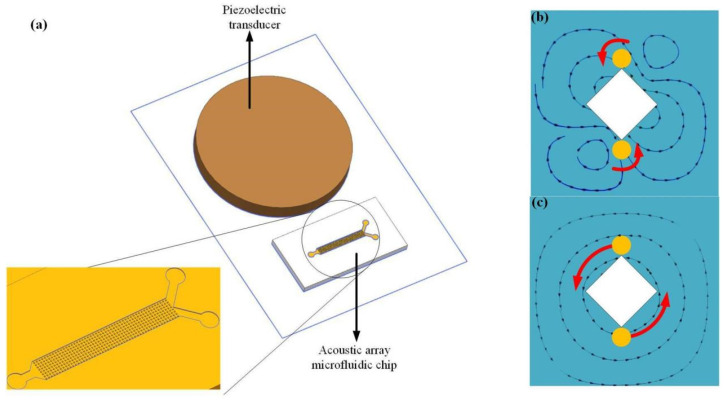
Schematic diagram of cell manipulation based on microfluidic chips. (**a**) Overall structure diagram of AMPA chip. (**b**) The process of cell self-rotation under the action of acoustic flow generated by microstructure vibration. Cells (orange) rotate in a certain direction (red arrows) under the effect of the acoustic streaming field (black arrows) generated by the rhombic micropillars (white squares). (**c**) The process of cell rotation around the microcolumn under the action of acoustic flow generated by microstructure vibration.

As shown in Figure 1a, the AMPA chip (34 mm in length, 18 mm in width) consists of microchannels molded from polydimethylsiloxane (PDMS), PDMS cover plates used to seal the microchannels, PZT (50 × 4mm) used to generate bulk acoustic waves, and glass sheets used to transmit bulk acoustic waves and as substrates. Among them, the side length of the rhombic microcolumns is 100 μm, and the spacing between the microcolumns is 100 μm. The dual-inlet design can effectively reduce the air intake channel and minimize the loss of valuable reagents.

In practice, the signal source causes the piezoelectric transducer to vibrate by giving it a signal of a certain frequency and voltage, which is then transmitted through a thin glass plate to the microcolumn in the microchannel, causing it to vibrate and generate an acoustic flow. The x and y direction velocity components of the vibration of the microcolumn can be expressed as follows [56]:

(1a) $$V_{x}=\omega d_{x}e^{i\omega t}$$

(1b) $$V_{y}=-i\omega d_{y}e^{i\omega t}$$

In the formula, $V_{x}$, and $V_{y}$ represents the velocity of the microcolumn in the x and y directions, ω is the vibration frequency, $d_{x}$ and $d_{y}$ represents the amplitude of the vibration in both the x and y directions. Different vibration amplitudes in the x and y directions can generate different vibration modes, forming different flow fields to rotate and manipulate cells. The specific relationship is presented in Section 3.

### 2.2. Sample Preparation

A suspension of monodisperse polystyrene microspheres with a diameter of 5 µm (5 mL, 1 wt%, Shanghai Yiyuan Biotechnology Co., Ltd., Shanghai, China) was added to Tween 20 at a concentration of 0.3% *v*/*v* (BioFroxx, Beijing, China) and vibrated for 30 s on a vibrator to mix the solution well. Optimize the parameters (voltage, frequency) of the acoustic flow-controlled system. Fluorescence experiments characterized microstructurally induced acoustic flows using 2 µm monodisperse fluorescent functionalized microspheres (0.5 mL, 2.5 wt%, Shanghai Yiyuan Biotechnology Co., Ltd., Shanghai, China). Adding Tween 20 prevents particle aggregation or adhesion to the channel.

Human breast cancer cells (MDA-MB-231) were purchased from the National Experimental Cell Resource Sharing Platform. The cancer cells were cultured in DMEM medium (Gibco, Carlsbad, CA, USA) supplemented with 10% fetal bovine serum (Gibco, Carlsbad, CA, USA), 1% penicillin–streptomycin (PS) (Solarbio, Beijing, China), and 1% glutamine, in a 5% ${CO}_{2}$ incubator at 37 °C. Before the experiment, the cancer cells were diluted to a concentration of 3.5 × 10^4^ cells/mL and washed twice with PBS (Solarbio, Beijing, China) to remove the residual serum. Finally, degassing treatment was performed to minimize the damage to the cells caused by cavitation.

The cell activity of the collected cancer cells was detected using a double staining technique-fluorescein diacetate (FDA) and propidium iodide (PI). FDA can freely enter living cells and be broken down by intracellular lipase to produce polar fluorescein, which emits green fluorescence in living cells. PI can only pass through the damaged cell membranes of dead cells, staining the DNA and emitting red fluorescence. Staining working solution preparation: (1) FDA stock: Add 4.16 mg of FDA powder to 100 µL of DMSO, shake or gently shake until completely dissolved to obtain an FDA stock of 1 mmol/L concentration; (2) PI stock: Add 1 mg of PI powder to 1 mL of DMSO, shake or gently shake until completely dissolved to obtain a PI stock with a concentration of 1.5 mmol/L; (3) Working solution preparation: Take 2 µL of the FDA stock and 4 µL of the PI stock and add them to 1 mL of PBS, gently shake until well combined. At this point, the concentration of the FDA solution is 2 µmol/L, and the concentration of the PI stock is 6 µmol/L, which is the final concentration of the working solution; (4) Storage of the stock solution: Due to the large amount of high-concentration FDA and PI stock solutions prepared, the volume of the working solution prepared is smaller. Therefore, they should be aliquot and stored separately in a refrigerator at −20 °C away from light. Before use, they should be stored in a refrigerator at 4 °C for thawing, with appropriate shaking during the thawing process. For better fluorescence, use only once after thawing.

### 2.3. Experimental Setup

**Figure 2:**
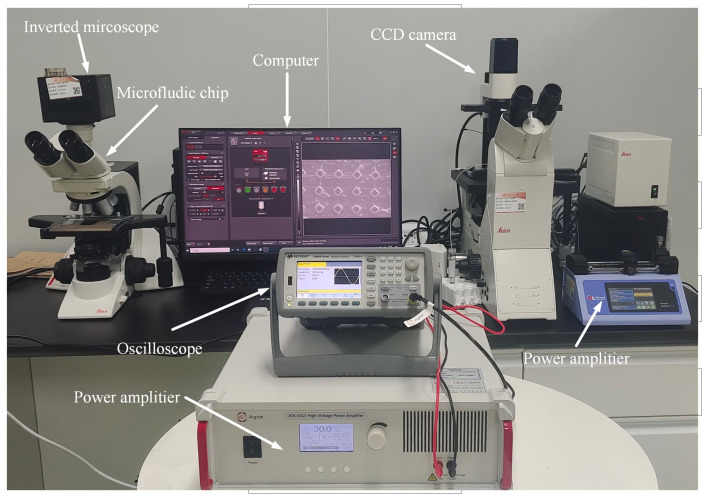
Design of the experimental platform.

As shown in Figure 2, the acoustic flow manipulation platform provides a stable periodic sinusoidal AC signal by a function signal generator (33500B, Keysight, Santa Rosa, CA, USA), which is amplified by a power amplifier (ATA-4315, Agitek, Xi’an, China) to increase the input power of the piezoelectric transducer. Meanwhile, an oscilloscope (UPO3204CS, UNI-T, Dongguan, Guangdong, China) was used to monitor voltage and frequency changes in real time during the experiment. The microfluidic chip was stably fixed on the stage of the microscope. Before running the chip, introduce anhydrous ethanol through the channel inlet into the microfluidic chip to enhance the hydrophilicity of the channel. To avoid the influence of residual alcohol on the experiment, rinse the PBS solution for 5 min. The solution containing the biological mixed sample was pumped into the microchannel by a precision flow pump (Pump 11 Pico Plus Elite, Harvard, Cambridge, MA, USA) through a transparent rubber tube (0.7 mm × 2 mm). The microstructure-induced acoustic flow patterns were visually characterized using an inverted fluorescence microscope (DMi8, Leica, Wetzlar, Germany). A charge-coupled device (CCD) camera (FASTCAM MINIUX100, Photron, Nagoya, Japan) connected to an optical microscope (DM2000, Leica, Germany) tracked the manipulation rotation process of cancer cells and polystyrene particles, respectively, and recorded the entire manipulation process. At the end of the particle and cancer cell manipulation experiments, the function signal generator was turned off, and ionized water was used to flush the manipulated cells out of the microchannel, based on the cell solution collected at the outlet. The total number of cells entering the microchannel can be obtained through a blood cell counter (Automatic Cell counter, C100, Reward Life Technology Co., Ltd., Shenzhen, China) for subsequent cell viability tests.

To characterize the cell activity, the collected cells are stained. The staining steps are as follows: (1) the cells collected from the outlet are first centrifuged; (2) wash the cells 2–3 times with PBS buffer and then add 200 µL of PBS to make a cell suspension; (3) take 100 µL of the prepared working solution and add it to 200 µL of cell suspension. Incubate the cells in the dark at 37 °C for 30 min; (4) finally, observe the live cells with green fluorescence at an excitation wavelength of 490 nm and the dead cells at an excitation wavelength of 535 nm, and then superposition the fluorescence images of the live cells with the images of the dead cells to show the final cell viability.

## 3. Experimental Study of Particle Manipulation Based on AMPA-Based Chips

### 3.1. Research on Different Vibration Modes

It is known from the experimental principle that different amplitudes of vibration will produce different vibration modes. In the actual experiment, the vibration of the microcolumn presents multiple modes, the most common of which are linear, quasi-circular, and elliptical vibrations. Linear vibration is when a vibration is applied in one direction and the vibration is stationary or minimal in the other direction. In this case, $d_{x}/d_{y}$ = 0$\;(d_{x}=0,d_{y}\neq 0).$ Circular vibration and elliptical vibration are applied in both x and y directions, with a phase difference of π/2, and different modes when the amplitude ratio is different. When $d_{x}{/d}_{y}$ = 1, it is a circular vibration mode, and when $d_{x}{/d}_{y}$ = 1/3, it is an elliptical vibration mode.

**Figure 3:**
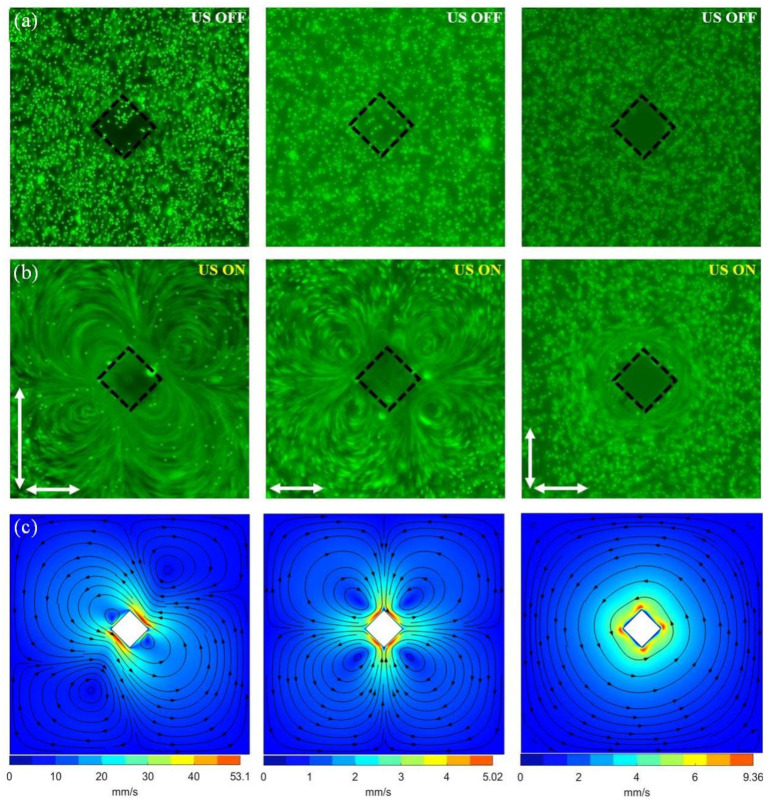
Results of acoustic streaming generated by ultrasonic-driven rhombic microstructures. Trajectories of fluorescent particles (5 µm) under the three corresponding vibration modes (elliptical, linear, and quasi-circular). (**a**) US device off. (**b**) US device on. The white arrow indicates the direction in which the vibration is applied. (**c**) Simulation of the acoustic streaming velocity distribution corresponding to different vibration modes. Black streamlines represent the shape of the acoustic streaming, and arrows indicate the direction of the acoustic streaming.

When the vibration modes are different, the distribution of acoustic flow generated around the microcolumn is also different. The elliptical vibration mode is composed of one main vortex and two secondary vortices, as shown in Figure 3a. The linear vibration mode generates four symmetrical micro-vortices, as illustrated in Figure 3b and Video S2. The quasi-circular vibration mode produces one large vortex, as depicted in Figure 3c. It can be known from the simulation diagram that the maximum acoustic flow velocity $V_{max}$ is the smallest under the linear vibration mode, the maximum sound flow velocity of the quasi-circular vibration mode is in the middle, while that of the elliptical vibration mode is the greatest. The three vibration modes are highly dependent on the resonant state of the entire chip and can match the corresponding resonances by changing the frequency. All three vibration modes can manipulate [57] particles, but since the elliptical vibration mode is the most common, subsequent studies have adopted the elliptical vibration mode.

In this study, COMSOL Multiphysics 6.2 was used for numerical solutions to effectively characterize the acoustic streaming distribution around the microstructure vibration. Under the action of acoustic streaming, particles were introduced into the microchannel, and the motion trajectories and forces of the particles suspended around the microstructure were solved. This solution process usually includes the following three steps [58]. The first step is to use the thermal-viscous acoustics module in the frequency domain to solve the control equations and obtain the first-order acoustic field. The second step is to use the laminar flow module, taking the first-order quantities as mass sources and volume forces, and solve the control equations to obtain the second-order acoustic field. The third step is to use the particle tracking module to study the motion of particles suspended around the microstructure in the acoustic streaming field. A simulation model was designed to study the acoustic streaming caused by the vibration of microcolumns. The particle tracking module was used to predict the motion trajectories of particles in the acoustic streaming field and further analyze the forces on the particles. The simulation domain was limited to a square box (500 µm × 500 µm), and the microstructure was defined as a rhombic microcolumn located in the middle of the square box. The COMSOL simulation has a mesh with a maximum element size of 13 μm, a minimum element size of 0.15 μm, a maximum element growth rate of 1.08, a curvature factor of 25, and a narrow region resolution of 1. The model consists of two parts: fluid and solid, where the fluid part uses water as the reference medium, with physical properties including a density of 997 kg/m^3^, a sound speed of 1496.73 m/s, a dynamic shear viscosity of 0.89 mPas, a bulk viscosity of 2.47 mPas, a thermal conductivity of 0.6075 W/m·K, a specific heat capacity of 4181.5 J/kg·K, a thermal expansion coefficient of 2.57 × 10^−4^ K^−1^, and a compressibility of 448 T/Pa. For the solid part, PDMS material is chosen, and in the model, it is assumed to be rigid, with its own deformation ignored. Regarding the boundary conditions, the microcolumn-shaped boundary is defined as the driving source, applying the vibration velocity boundary conditions as shown in Equations (1a) and (1b). Other boundaries are defined as constant temperature conditions. The density of the particles is 1060 kg/m^3^, and the sound speed is 2350 m/s. The operating parameters include a fundamental vibration frequency of 45 kHz. According to the research, the vibration of the microcolumn is mainly caused by the resonance frequency of the piezoelectric ceramic excited substrate. Therefore, the solid in the model is assumed to be rigid, and the deformation of the microcolumn itself is ignored. A vibration velocity was applied at the boundary of the microcolumn. Such a vibration boundary simulates the solid–liquid interface between PDMS and the surrounding medium without considering the material properties of PDMS. When different boundary conditions are applied, three vibration modes of straight line, ellipse, and circle can be obtained, and the generated acoustic streaming distributions are also different, as shown in the simulation diagram in Figure 3.

For the acoustic streaming velocity, based on the perturbation theory, the parameters in the field are decomposed into undisturbed, oscillating, and steady parts. The time-averaged acoustic streaming velocity is obtained by solving the second-order continuity equation and the Navier–Stokes equation. The relevant formula is [59] as follows:

(2a) $$\rho_{0}\nabla \cdot \langle v_{2}\rangle =-\nabla \cdot \langle \rho_{1}v_{1}\rangle$$

(2b) $$\eta \nabla^{2}\left\langle v_{2}\right\rangle +\beta \eta \nabla \left(\nabla \cdot \left\langle v_{2}\right\rangle \right)-\left\langle \nabla p_{2}\right\rangle =\left\langle \rho_{1}\frac{\partial v_{1}}{\partial t}\right\rangle +\rho_{0}\langle \left(v_{1}\cdot \nabla \right)v_{1}\rangle$$

In the formula, $\rho_{0}$ is the fluid density. ⟨...⟩ represents the average time value over the entire oscillation period. $v_{1}$ represents the acoustic streaming velocity in the first-order field, and $v_{2}$ represents the acoustic streaming velocity in the second-order field. η is the dynamic shear viscosity. β is the viscosity ratio. $p_{2}$ is a second-order stress tensor. *t* is the time.

Among them, $\left\langle v_{2}\right\rangle$ is solved by the first-order field parameters $v_{1}$ (first-order sound velocity) and $\rho_{1}$ (first-order density perturbation).

The motion of the particle velocity is jointly determined by the acoustic radiation force and the acoustic streaming-induced drag. In a standing wave sound field, the acoustic radiation force acting on a compressible, spherical particle in a viscous fluid is as follows:

(2c) $$F_{rad}=-\nabla U_{rad}$$

(2d) $$U_{rad}=\frac{4\pi }{3}r^{3}\left(f_{1}\frac{\kappa_{0}}{2}p_{in}^{2}-f_{r2}\frac{3\rho_{0}}{4}v_{in}^{2}\right)$$

(2e) $$f_{1}\left(\tilde{\kappa }\right)=1-\tilde{\kappa }$$

(2f) $$\tilde{\kappa }=\frac{\kappa_{p}}{\kappa_{0}}$$

(2g) $$f_{2}\left(\tilde{\rho },\tilde{\delta }\right)=\frac{2\left[1-\gamma \left(\tilde{\delta }\right)\right]\left(\tilde{\rho }-1\right)}{2\tilde{\rho }+1-3\gamma \left(\tilde{\delta }\right)}$$

(2h) $$\gamma \left(\tilde{\delta }\right)=-\frac{3}{2}Re\left[\frac{1+i\left(1+\tilde{\delta }\right)}{\tilde{\delta }}\right]$$

In the formula, $U_{rad}$ is the potential energy of acoustic radiation, r is the radius of the particle, $k_{0},k_{p}$ respectively, are the compression rates of the fluid and the particle, $f_{1}$ is the unipolar scattering coefficient, $f_{2}$ is the dipole scattering coefficient, $p_{in},v_{in}$ respectively, are the pressure and velocity of the incident sound wave, $and\;\tilde{\rho }=\frac{\rho_{p}}{\rho_{0}}$ is the ratio of the density of the particle to that of the fluid. $\tilde{\delta }=\frac{\delta }{r}$, it is the ratio of the thickness of the viscous boundary layer to the radius of the particle.

The formula for calculating the acoustic streaming-induced drag $F_{d}$ is as follows:

(2i) $$F_{d}=6\pi a\eta \left(\langle v_{2}\rangle -u\right)$$

In the formula, a represents the particle radius, u is the velocity of the particle.

The motion of particles follows Newton’s laws of motion: $m_{j}\frac{dv_{j}}{dt}=\sum F_{i}\left(r_{j}\right)$, that is, the particle’s acceleration is determined by the resultant force it experiences, and from this, the change in its velocity can be derived.

### 3.2. Research on the Effects of Frequency and Voltage on the Rotational Speed of a Single Particle

Experiments show that adjusting the frequency over a wide range would change the vibration mode of the acoustic flow, but changing it over a small range would only affect the rotational speed of the particles. Therefore, the same voltage (30 Vpp) was maintained in different vibration modes, and the effect of the power supply frequency on the rotational speed was measured by adjusting the frequency over a small range. And the experiment found that the power supply voltage also affects the rotational speed of the cells, so keeping the frequency constant in different vibration modes investigated the effect of the power supply voltage on rotational speed by adjusting the power supply voltage from 0 to 60 VPP, with a step of 10 VPP each time.

**Figure 4:**
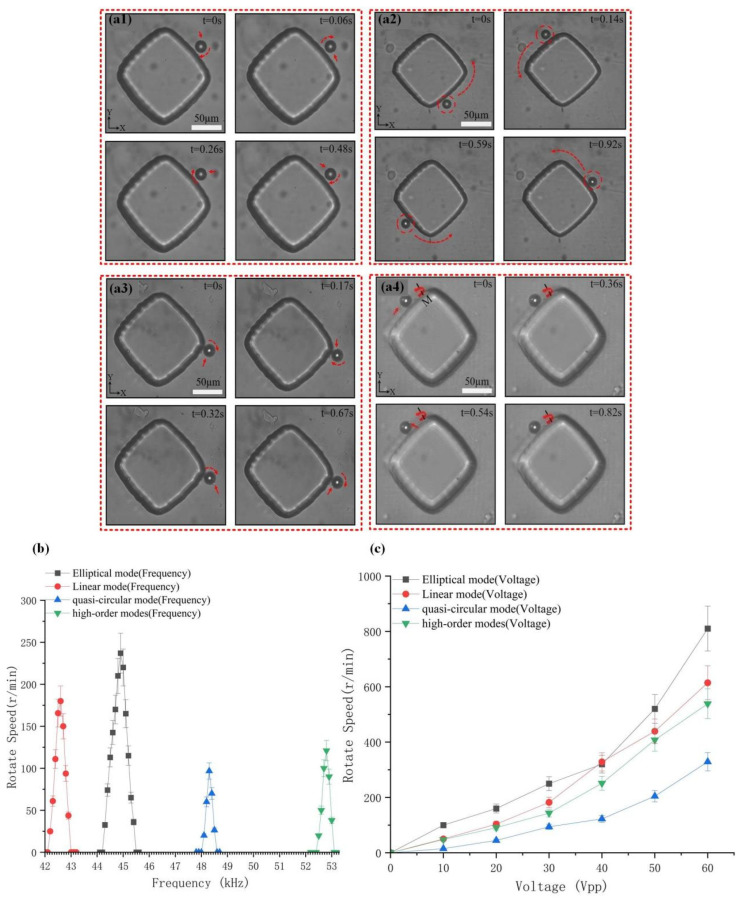
Research on the rotation operation of particles and the optimization of manipulation frequency and voltage. (**a1**–**a4**) Schematic diagrams of particle rotation under elliptical, quasi-circular, linear, and high-order vibration modes. The cells are indicated by red arrows, and the rotation direction is marked by red dashed arrows. (**b**) Relationship between rotational speed and frequency, and voltage for each vibration mode. (**c**) Relationship between rotational speed and voltage for each vibration mode.

When the driving frequency is set to 45 kHz, the system forms an elliptical vibration mode, pumping 5 µm particles into the microchannel. After the piezoelectric transducer is activated to generate ultrasonic waves (US), the particles are captured on the microstructure and rotate rapidly around the Z-axis under the action of acoustic vortices (Figure 4(a1) and Video S1). The rotation direction is indicated by the red dashed arrow. This rotation is driven by the combined effect of acoustic radiation force and acoustic streaming-induced drag torque: the acoustic radiation force captures the particles through the acoustic pressure gradient, while the drag torque generated by the acoustic streaming on the microstructure surface drives the rotation, enabling the particles to stably suspend and freely rotate in the plane. Experiments reveal that the rotational angular velocity of the particles is highly sensitive to frequency changes, showing a nonlinear response characteristic (Figure 4b). When the frequency increases from 44.9 kHz to 45.5 kHz, the angular velocity first reaches a peak of 238 r/min and then drops sharply to zero. This change is attributed to the transformation of the efficiency of acoustic wave energy transfer at 44.9 kHz, the optimal resonant state is achieved, but beyond this frequency, energy loss intensifies, leading to a decline in driving force until rotation ceases.

When the driving frequency is adjusted to 42.8 kHz, the system enters a linear vibration mode. At this point, the particles are captured by the US device and rotate rapidly within the plane (Figure 4(a2) and Video S1). The rotational angular velocity ω reaches a peak of 180 r/min at 42.6 kHz, but drops to 0 r/min at 43.0 kHz. When the frequency is increased to 48.2 kHz, the system switches to a quasi-circular vibration mode. The particles are no longer captured but orbit around the microcolumn driven by the acoustic flow (Figure 4(a3) and Video S1). The orbital angular velocity $\omega_{g}$ reaches a maximum of 96 r/min at 48.3 kHz but drops to 0 r/min when the frequency exceeds 48.6 kHz. Further increasing the frequency to 52.8 kHz, the system excites a higher-order vibration mode, and the particles rotate around the M-axis outside the YZ plane (Figure 4(a4) and Video S1). Higher-order vibration modes, due to the complex amplitude combinations of microcolumn vibrations in multiple directions, form acoustic flow fields with more complex spatial distributions, generating moments along non-traditional rotational axes (such as the M-axis), and ultimately achieving rotation in special dimensions. The rotational angular velocity reaches a peak of 120 r/min at 52.8 kHz, but drops to 0 r/min at 53.1 kHz. These results indicate that there are significant differences in the energy transfer efficiency of the acoustic field under different vibration modes, and the frequency variation can precisely control the motion mode (self-rotation, orbital rotation, or out-of-plane rotation) and rotational speed of the particles.

To evaluate the effect of the driving voltage of the US device on the rotational angular velocity of the particles in different vibration modes, repeated experiments were conducted using 5 µm diameter polystyrene spherical particles at the optimal frequency in different modes. The 5 µm particles were first pumped into the microchannel, with the particles randomly distributed around the microcolumn. Then, the US device was activated, the particles were rapidly captured and began to rotate, and the voltage was gradually increased from 0 to 60 Vpp, with a step size of 10 Vpp. Adjust the drive frequency to 44.9 kHz to form an elliptical vibration mode, where the rotational angular velocity of the particles increases rapidly with the increase in the drive voltage up to 800 r/min; adjust the drive frequency to 42.6 kHz to form a linear vibration mode in which the rotational angular velocity of the particles increases rapidly with the increase in the drive voltage, up to 620 r/min; adjust the drive frequency to 42.6 kHz to form a quasi-circular vibration mode, in which the rotational angular velocity of the particles increases rapidly with the increase in the drive voltage, up to 328 r/min; adjust the drive frequency to 52.8 kHz to form a high-order vibration mode, in which the rotational angular velocity of the particles increases rapidly with the increase in the drive voltage, up to 550 r/min. The relationship between the driving voltages of various vibration modes and the rotational angular velocity of the particles is shown in Figure 4c.

### 3.3. Multi-Particle Rotation Experiment

**Figure 5:**
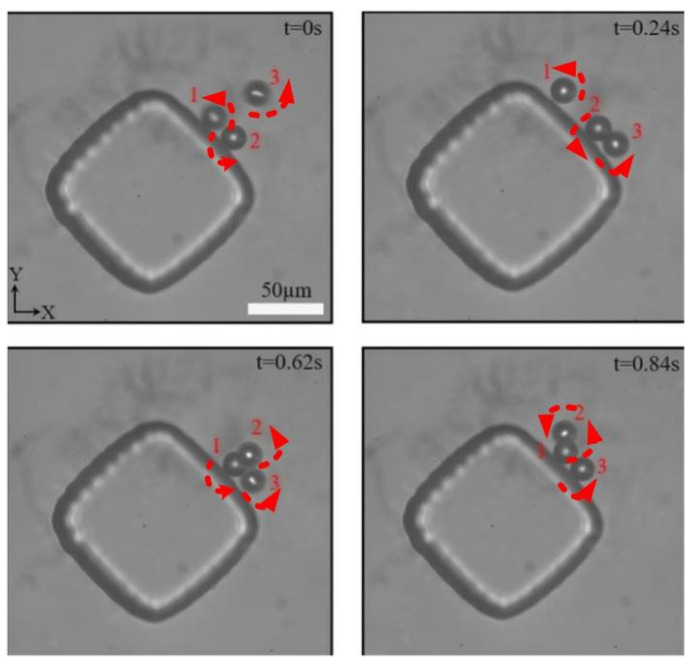
Rotational operation of multiple particles. Under the excitation of 45.5 kHz and 60 Vpp, three particles (marked with red numbers) rotate along the acoustic streaming direction (indicated by the red dashed arrow).

In addition, experiments have found that this method can not only manipulate a single particle, but also multiple particles simultaneously. As shown in Figure 5, multiple particles were captured simultaneously around the microcolumn, and it was observed that these particles were rotating in an orderly manner in a local area of the microcolumn. At different times, we observed three particles in different positions but still in a captured state. It is notable that when a microcolumn captures a particle, it is able to firmly hold the position of that particle. Once other particles enter the acoustic flow trap, the captured particle is no longer so firm and begins to spin irregularly in a local area. This suggests that the microcolumn has a certain dynamic regulatory mechanism for capturing and controlling different particles, which may change due to the influence of nearby particles.

### 3.4. Rotational Manipulation of Cancer Cells

Understanding the in-plane and out-of-plane rotation of cells can provide a deeper insight into cell behavior and the micro-environment, enabling better extraction of the morphological characteristics of cancer cells and facilitating the analysis of heterogeneity among cancer cells. Through the study of particle rotation, it was discovered that there are different vibration patterns at different frequencies. Therefore, for subsequent cancer cell rotation experiments, 44.9 kHz and 52.8 kHz were selected as the driving frequencies, respectively, to achieve in-plane and out-of-plane rotations of cancer cells.

**Figure 6:**
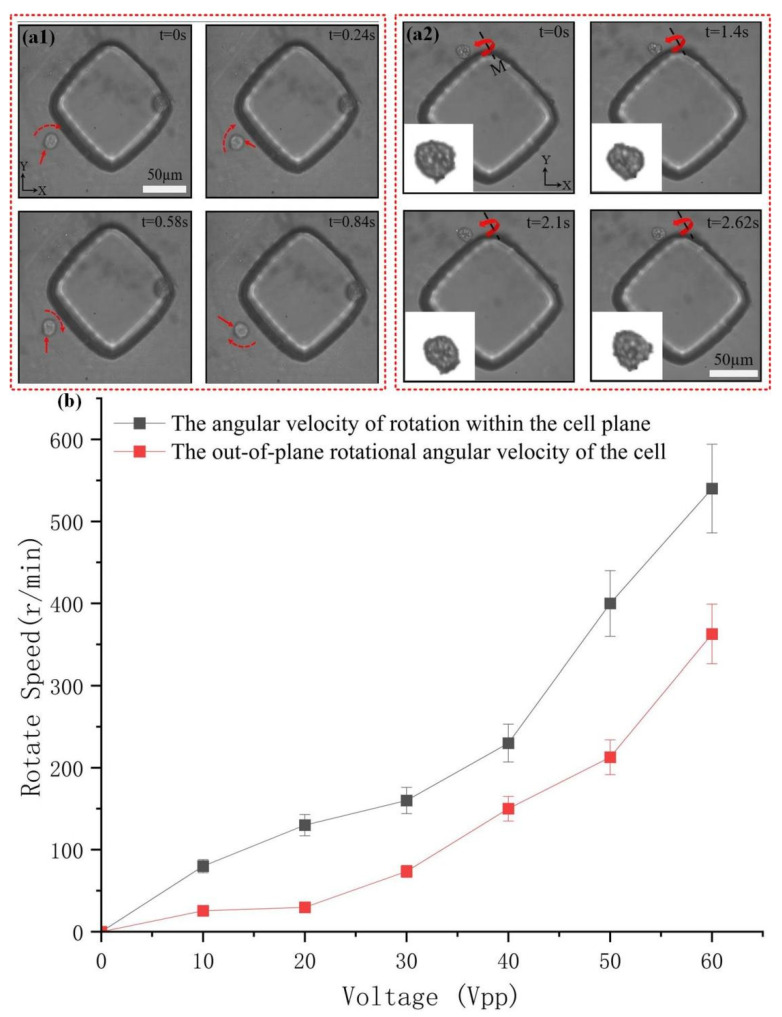
Research on cell rotation operation and optimal control voltage, (**a1**,**a2**) Schematic diagram of the rotation of cancer cells in the plane and out of the plane. The cells are indicated by red arrows, and the rotation direction is marked by red dashed arrows. (**b**) Relationship diagram between voltage and the angular velocity of cancer cells’ rotation in the plane and out of the plane at the optimal frequency of the elliptical vibration mode.

First, the cancer cells were inserted into the microchannel and randomly distributed around the microstructure. Then, the US device was turned on, and the cancer cells were rapidly captured and began to rotate under torque. The cancer cells are captured around the rhombic microcolumn at different times and rotate continuously in the plane along the Z-axis under torque, as shown in Figure 6(a1) and Video S3.

Subsequently, the frequency of the US device was adjusted to 52.8 kHz to enable the out-of-plane rotation of the cancer cells. The cancer cells were captured around the rhombic microcolumn at different times and rotated out of plane along the M-axis under torque, as shown in Figure 6(a2) and Video S3. Adjust the input voltage at the same drive frequency for both vibration modes, gradually increasing the voltage from 0 to 60 Vpp, with a step size of 10 Vpp. The relationship between the drive voltage and the rotational angular velocity of the cancer cells outside the plane along the m-axis as they rotate in the plane is shown in Figure 6b. With the increase in the drive voltage, the capture efficiency of the cancer cells increases rapidly. The maximum rotational speed of the cancer cells is 540 r/min when rotating in the plane and 360 r/min when rotating outside the plane. Both are slower than particles under the same conditions because the surface roughness of cancer cells is higher and their shape is irregular, resulting in a lower rotational speed compared to particles.

### 3.5. Effects of Biological Activity

To test whether the acoustic flow had an effect on cell activity, fluorescein diacetate (FDA) and propidium iodide (PI) were used to detect the cell activity of cancer cells collected at the outlet after the rinse was controlled. Based on the cell viability assay method presented in Section 2, the cell viability was tested. The cancer cells used in the experiment were diluted to a concentration of 3.5 × 10^4^ cells/mL. The final result was that in the experimental group, there were a total of 83 cells under bright field, and after FDA-PI staining, 82 cells were found to be viable and 1 cell was dead; in the control group, there were a total of 38 cells under bright field, and after staining, all cells were found to be viable.

**Figure 7:**
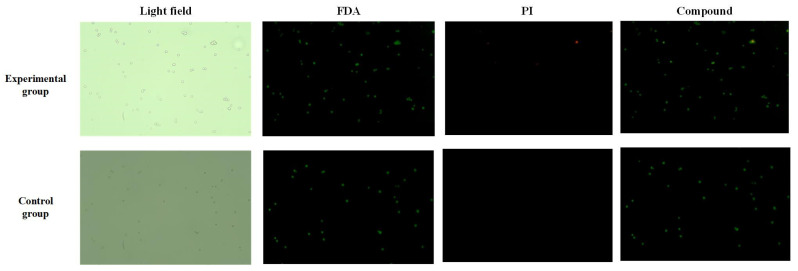
Post-manipulation activity detection of cells (Magnification: 10 times).

Figure 7 shows the fluorescence of collected cancer cells after 30 min of treatment under ultrasound compared with that of untreated cancer cells (control group). Green fluorescence represents FDA fluorescence, and red fluorescence represents PI fluorescence. In the superimposed results of FDA and PI, most untreated cancer cells (98%) still showed green fluorescence, while red fluorescence was almost zero. Most of the acoustically treated cancer cells (94%) still showed green fluorescence compared with the control group. This result suggests that vibration-induced acoustic flows from microstructures can gently rotate and manipulate cancer cells without causing damage to the cells.

## 4. Conclusions

In this work, we propose a non-invasive three-dimensional cell manipulation method based on acoustic microfluidic chips and study the acoustic field conditions of four vibration forms of micropillars. We investigated the rotation status of the particles in these four vibration modes and the relationship between rotational speed and frequency, and voltage. Subsequently, we conducted manipulation experiments on cells and studied the effect of driving voltage on rotational speed at the optimal vibration frequency. In addition, we evaluated the activity of the cells after manipulation, addressing the problem that most existing manipulation methods cause damage to the cells. Although we conducted manipulation experiments on cells, the actual size and shape of the cells have an impact on the manipulation results, which makes precise manipulation of cells difficult in some tasks. Therefore, improving the precision of manipulation is an essential direction for future development. This manipulation method has the characteristic of being directionless in rotational direction control. Because under the same power and voltage conditions, regardless of how the positive and negative poles of the power amplifier are connected to the positive and negative poles of the piezoelectric transducer, the acoustic flow field generated by the microcolumn is consistent in both direction and magnitude. This leads to the fact that the rotational direction of the particles or cells will not change. Therefore, at present, this method cannot achieve active control of the rotational direction. The current data only represents this trial. Further trials will be conducted in the future to expand the data volume and conduct more comprehensive verification.

## Data Availability

The raw data supporting the conclusions of this article will be made available by the authors on request.

## Supplementary Materials

The following supporting information can be downloaded at: https://www.mdpi.com/article/10.3390/mi16091068/s1. Video S1: Manipulation of 5 µm polystyrene particles under four vibration modes; Video S2: Visualization of acoustic streaming fields with different vibration modes using 5 µm fluorescent particles; Video S3: Visualization of in-plane and out-of-plane rotation of human breast cancer cells.
